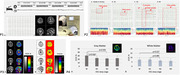# Impact of Sleep on Extracellular Volume in Human Brains: A Study with Simultaneous Sodium (^23^Na) MRI and EEG

**DOI:** 10.1002/alz.089650

**Published:** 2025-01-09

**Authors:** Xingye Chen, Ying‐Chia Lin, Nahbila‐Malikha Kumbella, Simon Henin, Zena Rockowitz, Anli Liu, Arjun V. Masurkar, James Babb, Yulin Ge, Yvonne W. Lui, Yongxian Qian

**Affiliations:** ^1^ New York University Grossman School of Medicine, New York, NY USA; ^2^ NYU Grossman School of Medicine, New York, NY USA

## Abstract

**Background:**

Alzheimer's disease is associated with neurotoxic amyloid‐beta (Aβ) plaques. Studies in mice demonstrated that cerebrospinal fluid (CSF) clearance, if impaired, reduces Aβ clearance by 70% and that sleep enhances CSF clearance via expanding extracellular space by 60%. However, the impact of sleep on extracellular volume in human remains unclear due to lack of non‐invasive technology. In this study, we use unique sodium (^23^Na) magnetic resonance imaging (MRI) to measure extracellular volume fraction (ECVF) in healthy subjects while monitoring their sleep stage with MRI‐compatible electroencephalography (EEG).

**Method:**

With IRB approval, we studied 16 cognitively‐healthy subjects (age 52.9 ± 17.6 years, ranging 27–77 years, 7 males and 9 females). The study lasted 1.5 hours, including four repeated 16‐min sodium MRIs on a clinical scanner at 3T (Prisma, Siemens), and continuous EEG recording (Brain Vision, 32‐channel, Garner, NC). The subjects were instructed to relax and fall asleep. Sleep was scored to five stages (wake, N1, N2, N3, and REM), based on American Academy of Sleep Medicine (AASM) Manual (v2.6, 2020). Sodium MRI was performed with a dual‐tuned (^1^H‐^23^Na) birdcage coil (QED, Cleveland, OH) and a custom‐developed pulse sequence, twisted projection imaging (TPI), with parameters: FOV=220mm, matrix size=64, 3D isotropic, TE/TR = 0.5/100ms, flip angle=90°, averages=6, and TA=16min. ECVF, *a*_e_, was calculated on the sodium images voxel‐by‐voxel, i.e., s = ΔV (a_e_C_e_ + a_i_C_i_) = ΔV (145 a_e_ + 15 a_i_), with a_e_ + a_i_ = 1.

**Result:**

In **panel P1** is the set‐up of the study, including the MRI, EEG, sodium images, and hardware set‐up. In **P2** are typical EEG waveforms and spectra of sleep stages. In **P3** is a representative of ECVF maps from a subject (43 years old, female). In **P4** are outcomes of the study. Surprisingly, we observed a decrease in ECVF with sleep stage in the gray matter (*P*=0.036) and in the white matter (*P*=0.085, nearly significant).

**Conclusion:**

This study surprisingly observed a decrease in ECVF with sleep stage in the gray matter of healthy subjects, and in white matter as possible. This finding is contrary to the outcome from the animal studies and needs to confirm with more human subjects.